# Emotion processing in depression and anxiety disorders in older adults: systematic review

**DOI:** 10.1192/bjo.2020.143

**Published:** 2020-12-03

**Authors:** Vanessa Gray, Katie M. Douglas, Richard J. Porter

**Affiliations:** Department of Psychological Medicine, University of Otago, Christchurch, New Zealand; Department of Psychological Medicine, University of Otago, Christchurch, New Zealand; Department of Psychological Medicine, University of Otago; and Canterbury District Health Board, Christchurch, New Zealand

**Keywords:** Emotion processing, older adults, cognition, late-life depression, late-life anxiety

## Abstract

**Background:**

Emotional cognition and effective interpretation of affective information is an important factor in social interactions and everyday functioning, and difficulties in these areas may contribute to aetiology and maintenance of mental health conditions. In younger people with depression and anxiety, research suggests significant alterations in behavioural and brain activation aspects of emotion processing, with a tendency to appraise neutral stimuli as negative and attend preferentially to negative stimuli. However, in ageing, research suggests that emotion processing becomes subject to a ‘positivity effect’, whereby older people attend more to positive than negative stimuli.

**Aims:**

This review examines data from studies of emotion processing in Late-Life Depression and Late-Life Anxiety to attempt to understand the significance of emotion processing variations in these conditions, and their interaction with changes in emotion processing that occur with ageing.

**Method:**

We conducted a systematic review following PRISMA guidelines. Articles that used an emotion-based processing task, examined older persons with depression or an anxiety disorder and included a healthy control group were included.

**Results:**

In Late-Life Depression, there is little consistent behavioural evidence of impaired emotion processing, but there is evidence of altered brain circuitry during these processes. In Late-Life Anxiety and Post-Traumatic Stress disorder, there is evidence of interference with processing of negative or threat-related words.

**Conclusions:**

How these findings fit with the positivity bias of ageing is not clear. Future research is required in larger groups, further examining the interaction between illness and age and the significance of age at disease onset.

Emotional cognition involves a range of functions, including ‘perceiving, interpreting, managing, and generating responses to socially relevant stimuli, such as intentions and behaviour of others’.^1^ Being able to undertake these functions efficiently is an important part of interpersonal relationships and social interactions. Interpretation of affective information is vital to these interactions as it influences emotional states and governs behavioural responses in social situations. Difficulties in social situations and avoidance of these, in the context of mental disorders, may maintain clinical levels of distress in patients and hinder attempts to treat their disorder. Further, emotional material can impair efficient cognitive processing by inducing biases in attention or decision-making, and by interfering with efficient cognitive processing, thereby impairing other aspects of cognition.

## Emotion Processing in Depression

The cognitive neuropsychological hypothesis of depression^2^ posits that in depression there is both behavioural and neurocircuitry evidence of a bias toward negative emotional stimuli. It is suggested that those who are more vulnerable to depression tend to perceive social cues as more negative and attend to, and recall more, negative information.^3^ These biases may play a role in precipitating depression, maintaining depression and conferring susceptibility to relapse. Antidepressants may reverse this bias relatively quickly, but it takes some time for this change to be translated, via improved social interactions, into a reduction in depressive symptoms.^4,5^ Multiple paradigms have been used to investigate emotion processing, and several, when studied in groups of younger individuals with depression, show differences broadly supporting the cognitive neuropsychological theory. For example, using a dot probe task, several studies have shown a bias toward sad faces compared with neutral faces in patients experiencing a major depressive episode.^6^ In addition, there is a bias in individuals with depression toward perceiving positive (happy), neutral or ambiguous facial expressions as more negative or less happy, compared with those in healthy control groups.^7,8^ In more complex social processing, examined with the Reading the Mind in the Eyes Task (RMET) and other complex tasks, people with depression perform significantly worse than control participants.^9^

## Emotion Processing in Anxiety

In younger adults with anxiety disorders, there is the tendency for a bias toward threat-related stimuli, which is hypothesised to maintain a heightened sense of threat. For example, in facial expression recognition (FER) tasks, evidence suggests that individuals with social anxiety are not significantly different from healthy controls in identifying facial expressions,^10,11^ but show a tendency to misidentify neutral facial expressions as angry.^12^ Attentional bias toward threat-related stimuli has been reported in Generalised Anxiety Disorder (GAD), when using a dot probe task.^13^ There is also evidence of increased response latencies to both anxiety-related words and generally negative words when using emotional Stroop (eStroop) tasks across anxiety disorders, suggesting an attentional bias toward threat-related stimuli.^14^ In younger adults, changes in emotion processing similar to those in other anxiety disorders have also been seen in Post-Traumatic Stress Disorder (PTSD). For example, a meta-analysis of eStroop task performance in PTSD^15^ found that individuals with PTSD, compared with healthy controls, showed impairments in the eStroop task when processing trauma-related and generally threatening, but not positive, information.

## Changes in Emotion Processing in Ageing

Multiple experimental paradigms examining different aspects of attention and memory, as well as meta-analysis of attention and memory in younger compared with older adults,^16^ have suggested a ‘positivity bias’ in older adults.^17^ For example, older participants have been shown to be more likely to remember positive images compared with negative images, whereas a preference for negative images was observed in young adults.^18^ This positivity bias has been demonstrated in multiple other emotion processing paradigms, including those examining autobiographical memory,^19,20^ working memory,^21^ attention to emotional faces^22^ and recall of facial expressions.^23^ Evidence also shows that older adults are slower and less accurate in studies investigating the effects of ageing on face perception by using tasks such as face detection,^24^ face identification^25,26^ and emotion recognition.^27–29^

Several theories attempt to explain this bias. Socioemotional selectivity theory, suggests that as time to the end of life becomes shorter, goals change focus and reflect a preference for emotional meaning and satisfaction.^30^ This change in goals then affects how we process emotions. An alternative theory is dynamic integration theory.^31^ Since processing of negative affect may be more cognitively demanding,^32^ this theory suggests that as we age, diminishing cognitive capacity (reduced capacity for processing) makes it more difficult to accept and integrate negative feelings, and therefore older adults disconnect from negative feelings, resulting in a positivity bias.^33^ However, if this theory is accurate then it is argued that this effect would be greatest in those with poorer or impaired cognitive function, whereas in fact, the opposite appears to be the case.^34,35^ Furthermore, depression is associated with cognitive impairment but, as noted, seems to increase bias for negative material – although not necessarily the integration and processing of this.^36^

A further theory, the aging brain model, suggests that the changes in emotional cognition seen in older adults may be associated with age-related changes in adrenergic and amygdala function,^37^ which leads to an impairment in the processing of negative stimuli. Studies do show differences in brain activation associated with emotion processing as ageing occurs, indirectly suggesting a decline in processing capacity in emotion processing areas. For example, older adults show reduced limbic and greater cortical activation (e.g. insula, frontal cortex) during processing of emotional faces.^38,39^ Some of these changes have been shown to correlate with the positivity bias. Sakaki et al,^40^ found increased negative coupling between the medial prefrontal cortex (MPFC) and amygdala and enhanced MPFC activity when learning emotional faces. This increase in MPFC activity may indicate an attempt to overcome an age related decline in capacity of MPFC processing areas. In general, a reduction in activity of prefrontal cortical processing areas, with concomitant increased limbic activation (amygdala, basal ganglia), has also been shown in studies of depression both in young and older individuals during emotion processing,^41^ in particular in response to sad faces.^42^

Research in Late-Life Depression (LLD) is further complicated by the issue of age at disease onset. Onset of depression at a later age is associated with greater neuropsychological abnormality, white matter hyperintensities and a higher rate of dementia at follow-up.^43–45^ The fact that late-onset depression has not been associated with frequent episodes across the lifespan may also be important in determining emotion processing. It may therefore be that emotion processing deficits, if they exist, are different between early- and late-onset depression.

To date, research examining emotion processing in older adults with mood and anxiety disorders has not been systematically synthesised. This review aims to examine this literature to understand the interactions between depression and anxiety disorders, cognitive decline and the positivity bias in older adults, and their effects on emotion processing.

Based on the factors discussed above, our questions were as follows:

- In LLD, Late-Life Anxiety (LLA) and PTSD, does the positivity bias in old age mitigate the emotion processing abnormalities that might be expected given the evidence of negativity bias in younger people with depression or anxiety disorders?
- Are changes in emotion processing circuitry in LLD, LLA and PTSD similar to those seen in younger people with depression or anxiety?
- Are behavioural and brain changes different in people with late-onset depression compared with early-onset depression, and what is the relationship between these changes and age-related cognitive decline?

## Method

### Protocol and registration

Details of the protocol for this systematic review were registered with the Prospective Register of Systematic Reviews (registration identifier: CRD42020124980; accessed at: https://www.crd.york.ac.uk/prospero/display_record.php?ID=CRD42020124980).

### Search strategy

Up to December 2019, a systematic review of electronic databases was carried out for relevant papers, using PubMed and Web of Science. In the initial search, the search terms used were ‘depression’, ‘anxiety’, ‘PTSD’, ‘bipolar’, ‘emotion processing’, ‘older persons’ and ‘elderly’, in different permutations. Reference lists of all relevant papers were then checked to ensure inclusion of all pertinent articles. Citations of relevant articles were then followed using Web of Science to allow for capture of any missed articles.

### Inclusion criteria

Peer-reviewed articles involving assessment with an emotion-based processing task and comparison of a clinical sample with a healthy control sample were included in the review. Studies examining all psychiatric disorders were to be considered; however, studies were only found for depression, anxiety and PTSD populations. Sample participants were to be aged over 60 years and samples were to be categorised as ‘older adult’ or similar.

### Exclusion criteria

Reasons for exclusion were comorbid major medical or neurological disorder in either group in the study, and studies involving participants with mild or greater levels of cognitive impairment (Mini-Mental State Examination score <25 or equivalent). All studies were limited to English-language publications.

### Full study review

This review was undertaken with recommended Preferred Reporting Items for Systematic Reviews and Meta-Analyses (PRISMA) guidelines and using the PRISMA statement to guide the search, screening and extraction process.^46^ Articles were screened by one of the reviewers, who independently reviewed the titles and abstracts of studies, to accept or reject for full-text review. The same reviewer then examined the full texts of the studies that had passed initial screening, to determine if they still met inclusion criteria. If inclusion of a paper was unclear, all three co-authors discussed this, to achieve a consensus. For each study, the following data was extracted: characteristics of the sample, including sample size, average age and baseline depression/anxiety severity; study design; cognitive tests used during assessment and study outcomes.

## Results

**Fig. 1:**
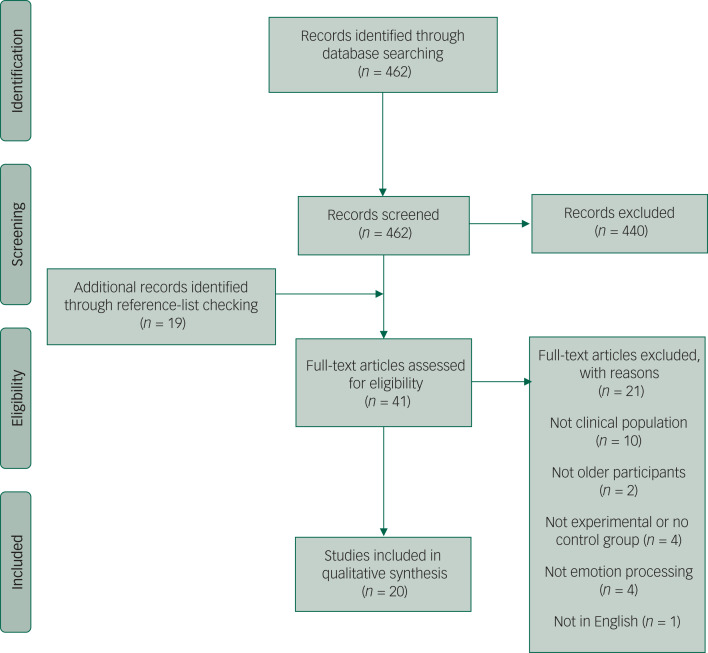
Preferred Reporting Items for Systematic Reviews and Meta-Analyses diagram of studies retrieved for the review.

The initial search for this review found 462 articles (Fig. 1). After a title and abstract review, 440 of these were excluded. The full text of the remaining 22 studies were obtained and reviewed. Nineteen additional papers were found through examination of the reference sections of the full-text studies. Of these 41 studies, 21 were then excluded for not being clinical or experimental studies, not including older participants or emotion processing, or not being in English. The remaining 20 papers are reviewed as follows. All of the studies included clinically diagnosed populations, unless otherwise stated. In this area of research, there is little consistency regarding terminology. LLD, depression and late- or early-onset depression are all used, with varying intended meaning; as such, we have deferred to the original authors and used the terminology that was present in the original article.

### Depression

Table 1 displays characteristics and main findings of studies examining behavioural data of samples with depression.

**Table 1:** Selected demographic characteristics of included studies examining behavioural data of samples with depression

Author	Arm	*N*	Gender	Age (s.d.)	Task	Outcomes
Dudley et al^47^	LLD	12	9 Female	74.4 (7)	Emotional Stroop (interference)	Participants with LLD had longer response times across blocks than control participants. Older participants with LLD showed a specific increase in response time to negative words.
	Control	12	9 Female	72.9 (8)		No difference in response time across any word valence.
Broomfield et al^48^	LLD	16	9 Female	73 (6.23)	Emotional Stroop (interference)	Older patients with LLD had lower reaction times across all trial types compared with controls. Older patients with LLD had significantly longer reaction times to negative words relative to neutral compared with controls.
	Control	19	9 Female	72.05 (5.56)		No change in reaction time for any valence of words.
Callahan and Hudon^49^	LLD	10	Not provided	Not provided	Emotional Stroop (interference)	Patients with LLD were slower than controls across all blocks.
	Control	15	Not provided	Not provided		Controls showed longer reaction times on negative compared with neutral blocks.
Huang et al^50^	LLD	55	38 Female	66.36 (5.42)	Emotional Stroop (interference)	Both positive and negative words were associated with longer latencies in both groups. There was, however, no group×emotion interaction on any of the measures.
	Control	40	25 Female	68.10 (5.30)		Control participants were more accurate and had shorter response latencies.
Mah and Pollock^51^	LLD	11	7 Female	73 (8.4)	Emotion regulation task (interference)	Overall response latencies did not differ between LLD and control groups. Difference was found for emotional stimuli compared with negative stimuli for the LLD group.
	Control	11	8 Female	75 (6.9)		Response latencies for all emotions were significantly longer compared with response to neutral stimuli.
Zhou et al^52^	LLD	14	9 Female	66.36 (5.20)	Primed face emotion recognition task (interference)	Reaction times for happy and sad primes were significantly longer than for ambiguous primes for all participants.
	Control	14	9 Female	65.64 (3.93)		
Zhou et al^52^ (Event-Related Potential)	LLD	14	9 Female	66.36 (5.20)	Primed face emotion recognition task (interference)	Participants with LLD had smaller event-related potential amplitudes overall, compared with controls for all priming stimuli, irrespective of valence. No differences were seen in event-related potential amplitudes between the different prime valences.
	Control	14	9 Female	65.64 (3.93)		No differences found between happy and ambiguous primes, but there were significant differences between happy (larger) and sad and ambiguous (larger) and sad primes within the control group.
Mah and Pollock^51^	LLD	11	7 Female		Emotion Perception task (recognition)	Participants with LLD were significantly more likely to misidentify a neutral face compared with control participants.
	Control	11	9 Female			
Savaskan et al^53^	LLD	18	14 Female	76.2 (1.8)	Face portrait recognition test (recognition)	Participants with LLD recalled fewer faces overall compared with controls. This lower recall was especially pronounced for recall of happy faces. There were no differences in recall for participants with LLD between happy and angry faces. Treatment with escitalopram did not improve memory recognition for happy faces, but did improve memory for faces with an angry expression.
	Control	22	16 Female	76.9 (1.8)		Participants in the control group had better memory for happy over angry faces.
Brassen et al^54^	LLD	13	13 Female	66.4 (6.1)	Verbal emotion recognition task (memory and recognition)	No differences in correct responding, reaction time or misattribution of emotions were seen between the LLD and control groups at baseline or follow-up.
	Control	13	13 Female	65.6 (6.1)		
Callahan et al^55^	LLD	19	15 Female	72.4 (9.0)	Memory for emotionally valenced words (memory)	At delayed recall, participants with LLD recalled significantly fewer words compared with control participants. At immediate recall, participants with LLD recalled more negative than neutral words. At delayed recall, participants with LLD recalled more positive and negative words than neutral words.
	Control	28	21 Female	72.1 (8.1)		No differences seen in immediate word recall between LLD and control groups. At both immediate and delayed recall control participants recalled more positive and negative words than neutral words overall.
Szanto et al^57^	Suicidal/LLD	24	38% Male	68.2 (8.7)	Reading the Mind in the Eyes Task (social cognition)	Participants with LLD who had attempted suicide had significantly poorer emotion recognition compared with controls. This difference was not maintained when global cognition was accounted for.
	Non-suicidal LLD	38	34% Male	70.2 (7.7)		Participants with LLD who had not attempted suicide had an emotion recognition ability, which fell between the control group and those with LLD who had attempted suicide, but was not significantly different from either.
	Control	28	61% Male	69.6 (6.3)		

LLD, Late-Life Depression.

#### Emotion interference

The emotion processing tasks discussed in this section involve inhibition of emotional information to carry out a cognitive task. This is most commonly a modified version of the Stroop paradigm (eStroop), which uses words that are of a positive, negative or neutral valence. Participants are required to name the colour of the presented word, but to ignore the word itself. A pilot study by Dudley et al^47^ (12 depressed, 12 controls) showed an interaction between group and word valence, which was explained by a greater interference (longer time to colour name) of depression-related words in the depressed group, with an effect size difference compared with controls of 0.9 (Cohen's *d*). Broomfield et al^48^ (16 depressed, 19 controls) also showed a group×valence interaction in participants with depression compared with controls, with participants with depression being slower to respond to negative words compared with neutral words. The group×valence interaction persisted when anxiety was controlled for. Using a similar eStroop paradigm, Callahan and Hudon^49^ found those with LLD (*n* = 10) were generally slower than controls (*n* = 15), but with no difference seen across valences. No group×valence analysis was reported. Finally, in 55 people with LLD and 40 controls, using an eStroop,^50^ controls were more accurate and had shorter latencies. There was no effect of emotion on accuracy, but there was on latency, with both positive and negative words associated with longer latencies. There was, however, no group×emotion interaction on any of the measures. Apart from the study by Dudley et al^47^ , no estimates of effect size difference between depressed and control groups are given.

Mah and Pollock^51^ used a facial emotion-based paradigm to measure emotion inhibition in 11 participants with depression and 11 controls. In this paradigm, participants were presented with faces displaying different emotions (happy, sad, fearful and neutral) and were asked to answer questions about a non-affective aspect of the face (inhibition). There was a significant group×valence interaction, whereby overall latencies were similar in both groups, but were significantly longer for all emotion-laden stimuli (not only negative emotions) compared with neutral stimuli in the control group and did not vary by emotion in the depressed group.

Zhou et al^52^ studied event-related potentials (ERP), in response to emotional faces, in 14 older adults with depressive symptoms (but specifically not meeting criteria for a DSM-IV diagnosis of depression), and compared this group with 14 controls. The study was included in the review, after discussion, since the patient group had significant depressive symptoms despite no formal diagnosis (Centre for Epidemiologic Studies Depression Scale mean group score of 20.21 ± 5.65). Participants were presented with an emotional prime (facial expressions: happy, sad, ambiguous) and then asked to identify the emotion of the target which followed. Older adults with depressive symptoms had smaller ERP amplitudes compared with controls, regardless of valence of the priming stimulus. Older adults with depressive symptoms showed no differences in amplitude between the different prime valences. In control participants, there were no differences found between happy and ambiguous primes, but there were significant differences between happy and sad (larger ERP) primes, and between ambiguous and sad (larger ERP) primes. However, it was notable that the group×prime interaction was not statistically significant (*p* = 0.07), making further analyses and conclusions very tentative. Behavioural data showed no differences between groups.

#### Emotion recognition and memory

Most commonly used is an FER task,^4^ in which participants are presented with various facial expressions (usually happy, sad, angry, disgusted, fearful, surprised and neutral) and asked to identify the emotion portrayed. Mah and Pollock^51^ used an FER task in which participants were presented with happy, sad, fearful or neutral faces. Although overall accuracy was similar between depressed (*n* = 11) and control (*n* = 11) groups, there was a significantly increased probability of participants with depression incorrectly identifying neutral faces.

Savaskan et al^53^ used a memory for faces paradigm that included both happy and angry faces. At baseline, patients with depression (*n* = 18) recalled fewer faces overall compared with controls (*n* = 22). There was no group×valence interaction, although the authors suggested that compared with controls, patients with depression showed lower ability to recognise previously viewed happy facial expressions after a delay. Following 4 weeks of treatment, the patient group showed a significant reduction in depressive symptoms and a significant improvement in general cognitive function. Further, their memory for angry faces significantly improved from baseline, whereas no effect was seen for happy faces.

Two studies examined emotion recognition and memory for emotional material, using verbal stimuli. Brassen et al^54^ used an emotion recognition task with positive, neutral and negative words to examine neural responses in 13 antidepressant-naïve female patients with LLD versus 13 controls. Participants were shown a positive, negative or neutral adjective, which was then replaced by a response screen where they indicated the valence of the word. No significant differences in response correctness, response time or misattributions of emotion were found at either time point between the two groups.

Callahan et al^55^ examined recall of neutral, positive and negative words from a list including 12 of each. At immediate recall, control participants (*n* = 28) displayed better recall of positive and negative words compared with neutral words, whereas participants with depression (*n* = 19) recalled more negative than neutral words. However, performance of the two groups was not directly compared. At delayed recall, both groups generally showed better recall of emotional compared with neutral words. During recognition, all participants were more likely to report false recognition for emotional than neutral words. Once again, there was no group comparison.

#### Social cognition

The RMET^56^ requires individuals to identify complex or social emotions from images portraying only the eyes of the face. Szanto et al^57^ examined the RMET in older adults with depression who had (*n* = 24) or had not (*n* = 38) attempted suicide. A control group was also examined (*n* = 28). Individuals who had attempted suicide performed significantly worse than healthy controls. The performance of participants with depression who had not attempted suicide fell between controls and those who had attempted suicide, but was not significantly different from either. Further analysis showed that when global cognitive function was accounted for (Mattis Dementia Rating Scale^58^), the significant difference found for individuals who had attempted suicide was not maintained, suggesting that the group's reduced ability to recognise social emotions may have been attributable to global cognitive impairment, rather than a specific impairment in social cognition.

#### Functional magnetic resonance imaging studies

Table 2 displays characteristics and main findings of studies examining neuroimaging data of samples with depression.

**Table 2:** Selected demographic characteristics of included studies examining neuroimaging data of samples with depression

Author	Arm	*N*	Gender	Age (s.d.)	Task	Outcomes
Aizenstein et al^59^	LLD	33	21 Female	67.7 (5.2)	Faces and Shapes task (recognition)	Greater limbic activation for participants with LLD than controls during affective processing
	Control	27	19 Female	71.6 (7.5)		
Huang et al^50^	LLD	55	38 Female	66.36 (5.42)	Emotional Stroop (interference)	LLD showed reduced activation in middle frontal gyrus and left dorsolateral prefrontal cortex, and increased activation in anterior cingulate cortex, compared with controls. This was mediated by cognitive reserve such that greater cognitive reserve correlated with greater middle frontal gyrus activation.
	Control	40	25 Female	68.10 (5.30)		
Brassen et al^54^	LLD	13	13 Female	66.4 (6.1)	Verbal emotion recognition task (memory and recognition)	Participants with LLD showed an attenuated neural response in the ventromedial prefrontal cortex in response to negative stimuli when compared with positive stimuli. At 7-month follow-up, when participants symptoms had significantly improved, this attenuated response was found to have normalised.
	Control	13	13 Female	65.6 (6.1)		
Briceño et al^60^	LLD	26	12 Female	Not provided	Emotion recognition task (recognition)	In older participants, women with LLD showed hypoactivation in emotion processing circuits, particularly the right prefrontal cortex, when compared with healthy controls. In contrast, older men with LLD showed hyperactivation in these areas when compared with the control group.
	Control	25	12 Female	Not provided		
Vanyukov et al^61^	LLD and suicide	18	6 Female	67.44 (7.0)	Face matching task (recognition)	LLD and suicide attempts did not predict a different response to angry faces than healthy controls.
	LLD	13	9 Female	68.15 (6.1)		
	Control	18	11 Female	70.05 (7.7)		

LLD, Late-Life Depression.

Aizenstein et al^59^ used a facial expression affective-reactivity task. Participants with depression (*n* = 33) showed greater subgenual cingulate activity during affective processing compared with controls (*n* = 27) with a significant correlation between white matter hyperintensity and activity. Huang et al^50^ (55 participants with LLD, 40 controls) showed reduced activation in middle frontal gyrus and left dorsolateral prefrontal cortex, and increased activation in anterior cingulate cortex, in the LLD group compared with controls in a study examining activation during an eStroop task. This was mediated by cognitive reserve such that greater cognitive reserve correlated with greater middle frontal gyrus activation in the LLD group. Brassen et al^54^ reported that in comparison with controls (*n* = 13), female patients with LLD (*n* = 13) showed attenuated neural response in the ventromedial prefrontal cortex in response to negative compared with positive words. When correlated with depression severity in the depressed group (Geriatric Depression Scale), reduced activation in the medial orbito-frontal cortex was correlated with higher depression scores. Increased activation in the superior medial frontal cortex, for positive compared with negative words, was also correlated with higher depression scores. At the 7-month follow-up, when patients’ symptoms had significantly improved, this attenuated response was found to have normalised.

Briceño et al^60^ examined the effects of age and gender on neural circuits in an FER paradigm that involved four emotions (happiness, sadness, fear and anger). The study included participants in younger and older age groups, as well as participants with and without depression (older participants with depression, *n* = 26; older controls, *n* = 25). When depressed and non-depressed groups were not separated by age and gender, no overall effects were detected between groups. However, when separated by age and gender, older women with depression showed hypoactivation in emotion processing circuits, particularly the right prefrontal cortex, when compared with older controls. In contrast, older males with depression showed hyperactivation in these areas when compared with the control group.

Vanyukov et al^61^ used the faces and shapes task,^62^ in which participants are required to match a target face to one of two presented faces. Authors used faces showing anger and fear during the face trials. In neutral trials, shapes were used as non-affective controls. The study examined patients with depression (*n* = 13), patients with depression who had attempted suicide (*n* = 18) and controls (*n* = 18). There was no difference in response to angry faces in either depressed group compared with controls. Responses to fear-related stimuli were not discussed.

### Anxiety disorders

Table 3 presents characteristics and key findings from reviewed studies examining samples with anxiety disorders.

**Table 3:** Selected demographic characteristics of included studies examining anxiety and post-traumatic stress disorder

Author	Arm	*N*	Gender	Age (s.d.)	Task	Outcomes
Price et al^63^ (Behavioural)	High (late-life GAD)	20	16 Female	67.2 (6.2)	Emotional Stroop (interference)	Reaction time for the threat related words was longer than neutral words. Reaction time for positive words was shorter than for neutral words.
	Mid	19	12 Female	68.5 (6.4)		Low- and medium-worry groups had faster reaction time for threat words compared with neutral, and slower reaction time for positive words compared with neutral.
	Low	21	15 Female	68.3 (5.6)		
Mohlman et al^65^ (Behavioural)	GAD	34	68% Female	66.67 (4.56)	Dot probe task (interference)	A bias away from positive information in positive-neutral pairs was seen across all participants.
	Control	28	71% Female	67.37 (5.53)		No significant differences in reaction time overall or between word types between the two groups.
Price et al^66^	GAD	16	11 Female	63.1 (3.1)	Emotional Stroop (interference)	Slower reaction times and decreased prefrontal cortex activation when identifying negative versus neutral words, compared with controls.
	Control	12	8 Female	67.2 (7.6)		
Wu et al^67^	GAD	16	9 Female	67.38 (5.78)	Faces and Shapes task (recognition)	No significant differences between the GAD group and the controls in either connectivity in the amygdala or BNST.
	Control	20	10 Female	67.8 (7.88)		
Karim et al^68^	GAD	17	10 Female	64 (6)	Faces and Shapes task (recognition)	No significant differences between GAD and controls in brain activation.
	Control	20	10 Female	67.5 (8.5)		
Wittekind et al^69^	PTSD	22	20 Female	72.73 (2.27)	Spatial cueing paradigm (interference)	No attentional bias for trauma-related stimuli in PTSD group.
	No PTSD	26	17 Female	73.00 (2.00)		
	Control	22	15 Female	73.73(2.98)		
Wittekind et al^70^	PTSD	20	18 Female	72.75 (2.31)	Emotional Stroop (interference)	PTSD group had longer response latencies to trauma and depression related words compared with healthy controls.
	No PTSD	26	17 Female	73.00 (2.00)		
	Control	21	15 Female	73.86 (2.99)		Within-group slowing for trauma compared with neutral words found in all groups in the study.

GAD, Generalised Anxiety Disorder; BNST, bed nucleus of the stria terminalis; PTSD, Post-Traumatic Stress Disorder.

Price et al^63^ divided 60 community recruited adults into high-, medium- and low-trait anxiety, based on responses to the Penn State Worry Questionnaire (PSWQ). A total of 90% of participants in the high-worry group (*n* = 20) scored ≥50 on the PSWQ, which has been noted as a cut-off for late-life GAD.^64^ The remaining participants (*n* = 40) formed comparison groups. An eStroop task containing positive, threat-related, and neutral words was used. In the high-worry group, response time for threat-related words was longer than neutral words (Cohen's *d* = 0.76). The high-worry group also showed faster reaction times for positive words than neutral. The low- and medium-worry groups showed the opposite pattern: faster response time for threat-related words compared with neutral, and slower response time for positive words compared with neutral. Between-group comparisons of estimated marginal means showed a difference between the high-worry and low-worry group, with bias toward threat-related words being greater (Cohen's *d* = 0.67).

Mohlman et al^65^ used a dot probe task which included depression, threat, positive and neutrally valenced words to examine attentional biases. There was no significant between-group differences in reaction time overall or between word types in GAD (*n* = 34) versus controls (*n* = 28). Using differences between probe-target congruent word pairs and incongruent pairs, bias scores were calculated. No significant differences in bias scores between GAD and control groups were found. A bias away from positive information in positive–neutral pairs was seen across all participants in the study. No significant change in performance following treatment (cognitive–behavioural therapy versus waitlist) was found.

#### Functional magnetic resonance imaging studies

Price et al^66^ examined functional magnetic resonance imaging responses to performance on an eStroop task in older adults with late-life GAD. Participants with GAD (*n* = 16) had slower reaction times when responding to negative versus neutral words, compared with controls (*n* = 12) (Cohen's *d* = 0.85), and also showed less activation in the prefrontal cortex in response to negative compared with neutral words. An increase in activation was seen in the GAD group in the left amygdala, compared with the control group.

Two studies^67,68^ examined functional connectivity associated with emotional reactivity in late-life GAD compared with healthy controls. Neither study found any significant differences between the GAD group and the controls, using a faces and shapes task. Both studies examined the data using the factor of worry, as measured by the PSWQ. Wu et al^67^ found that there was a significant interaction between group and ‘worry’ on connectivity between the left amygdala and left orbitofrontal cortex, MPFC and both anterior cingulate cortices, and on connectivity between the bed nucleus of the stria terminalis and the left orbitofrontal cortex. When worry was examined across groups, there was a U-shaped curve, whereby connectivity between limbic and cortical areas was optimal at medium levels of ‘worry’. The effect sizes of these curves varied from *r*^2^ = 0.21 to *r*^2^ = 0.25. Karim et al^68^ found that across the groups, increased global anxiety, measured by the Hamilton Anxiety and Depression Scale, was associated with greater activation in the parahippocampal area and precuneus. In contrast, worry (PSWQ) was associated with decreased precuneus and prefrontal activation. Complex mediation analyses broadly suggested that the mediation between increased white matter hyperintensity burden and anxiety symptoms are mediated by increased activation of limbic and paralimbic structures, and decreased activation of regulatory regions, such as the ventromedial prefrontal cortex.

### PTSD

One study, published as two papers, examined emotion processing in older populations with PTSD. Wittekind et al^69^ used a spatial cueing task involving priming with an emotional facial stimulus (anxiety, depression, trauma and neutral), followed by a spatial (left or right) decision, in response to a non-affective target (26 participants with PTSD, 22 controls). Authors found no attentional biases within the PTSD group for trauma-related stimuli. However, *post hoc* redistribution of participants into those who did or did not meet the criteria for depression showed that participants in the depressed sample had longer reaction times to depressive stimuli (Cohen's *d* = 1.5).

The same participants also completed an eStroop task^70^ containing words related to depression, trauma, anxiety or neutral words. Participants with PTSD showed longer response latencies to trauma- and depression-related words compared with healthy controls, but no differences in latency for neutral and anxious words. Slowing for trauma compared with neutral words was found across all groups in this study.

## Discussion

The main findings of the review were as follows. First, at a behavioural level, evidence regarding differences in emotion processing between individuals with LLD and controls is inconsistent. This is the case for interference of emotional material in cognitive processes (eStroop), memory for emotional compared with other material, and the explicit process of recognition of emotional facial expressions. Second, there are few studies in LLA. Studies suggest interference with processing by threat related words in anxiety and by trauma-related words in PTSD, but there are no replication studies. Finally, studies show differences in activation in emotional processing circuitry in LLD, with the general pattern of increased limbic but reduced prefrontal activity, as in younger individuals with depression.

The review more specifically examined three main questions as below.

### How does the positivity bias seen in older persons interact with biases toward negative or threat-related emotional material in depression or anxiety?

There are no consistent findings regarding any of the aspects of emotion processing studied. This was the case both for implicit processes (such as the eStroop) and for explicit processes (such as emotion recognition). Not all of these phenomena have been consistently replicated in younger people with depression either. For example, results on the eStroop have not been found to be consistent.^71^ The phenomenon seen most consistently in younger depression is the misinterpretation of, or attentional bias toward, neutral faces.^6,72^ In LLD, this was only examined in three studies and was not seen consistently.

The lack of consistent evidence of bias toward negative emotional stimuli in LLD may reflect a situation in which, on average, negative bias is less in LLD than in younger depression. It could be hypothesised that this relates to the positivity bias associated with ageing, which counteracts the biases seen in depression. However, the inconsistencies in the data are more likely to be related to the small numbers of studies and limited power of most studies. Further issues with the data are that there are no studies directly comparing emotion processing between younger patients with depression and LLD. Similarly no comparisons across the lifespan have been conducted in anxiety disorders. Therefore, the interaction of age with depression or anxiety cannot be fully evaluated. Finally, studies generally did not examine possible complicating factors, such as concomitant cognitive impairment, age at disease onset and the effects of medication.

### Are changes in emotion processing circuitry similar to those seen in younger people with depression or anxiety?

In younger people with depression, studies have generally shown a reduction in activity of dorsal-cognitive structures combined with increased activity of ventral-affective structures during emotion processing.^73^ The studies in LLD are broadly in line with this pattern with the largest studies showing increased activation of limbic structures or decreased activation of prefrontal structures in LLD compared with controls, in line with a general pattern of reduced top-down processing.^50,54,59^

Of interest, there was also evidence of an interaction between depression and both white matter lesions^59^ and cognitive reserve^50^ in determining patterns of activation during emotional processing. White matter changes were associated with an exaggeration of the increase in subgenual cingulate activation seen during emotion processing in LLD.^59^ In the study of Huang et al^50^, severity of depression was associated with reduced medial frontal activation during emotion processing, but this was attenuated by having greater cognitive reserve (measured using years of education and verbal fluency). Both findings suggest a situation in which if processing capacity is reduced for a variety of possible reasons, this may impair efficient emotion processing, resulting in processing being driven to a greater extent by limbic structures.

### Are behavioural and brain changes different in people with late-onset compared with early-onset depression, and what is the relationship between these changes and age-related cognitive decline?

Of note, our review excluded studies in which participants had a Mini-Mental State Examination score < 25. The rationale was that although we were interested in the interaction between mood and anxiety, cognitive ability and the relationship of these to emotion processing and positivity bias, we wanted to restrict the review to examining LLD and LLA and not to extend this into dementia. Four studies did examine the relationship between emotional processing and other aspects of cognitive functioning. Callahan et al^55^ examined the influence of depression on emotion processing in mild cognitive impairment (MCI), based on the suggestion that MCI plus depression is particularly likely to progress to dementia, and therefore constitutes a prognostically important subtype of MCI.^74,75^ Consistent with the hypothesis that negative information requires greater capacity to process, Callahan et al^55^ showed that for patients with MCI, immediate recall was better for positive words than negative words; however, this was not the case for MCI with depression, healthy controls or LLD. A caveat to this conclusion is the lack of an analysis of group×valence interaction. Furthermore, a similar effect was not seen in a separate examination of effects on an eStroop test.^49^ Although Huang et al^50^ did not find the hypothesised eStroop effect in LLD, there was more preserved middle frontal gyrus activity during eStroop performance in people with greater cognitive reserve. This suggests that cognitive reserve (measured using a combination of years of education and an executive task) mediates a more top-down emotional regulation, i.e. preserved processing capacity in those with greater cognitive reserve. Szanto et al^57^ examined social cognition in LLD. Those who attempted suicide had lower scores than controls but, interestingly, this did not survive covarying for general cognitive function, suggesting that the two functions are related, at least in depression.

### Specific issues in late-life anxiety disorders

Five studies examining emotion processing in LLA disorders were identified. Those using an eStroop task both showed increased latency for negatively valenced words.^63,66^ In one of the studies which examined brain activation,^66^ the hypothesised difference from controls was seen, with an increase in activation of part of the amygdala, accompanied by a decrease in activation of the dorsal lateral prefrontal cortex in older patients with GAD. Two further studies showed no difference between patients with GAD and controls,^67,68^ in brain activation and connectivity. However, in one there was a complex relationship between worry and connectivity, suggesting that connectivity between limbic and cortical areas was maximal at an intermediate level of worry.^66^ In the other study,^67^ anxiety was associated with greater activation in parahippocampal areas and precuneus. In general this is in keeping with attenuation of activity in, and connectivity with, processing areas seen in younger patients.^76^

Data regarding interference by trauma-related words in eStroop tasks has been consistently demonstrated in younger adults with PTSD.^15^ Both in LLA and PTSD, studies examining simple attention bias toward negative stimuli have not shown a difference from controls.^65,69^ This may suggest that the basic focus of attention is not altered, but that negative emotional stimuli do interfere with processing.

### Limitations and recommendations for future research

This review has limitations both directly related to the methodology and to the content of the studies reviewed. Related directly to the review, it considered English-language papers only. Although this is standard practice for an English-language review, it may mean that some relevant studies have been missed. Second, meta-analysis has not been possible given the heterogeneity of paradigms investigated in the studies examined, and in the variety of ways the data has been analysed and presented.

Limitations of the data, which can be translated into recommendations for the field are, as follows. First, although studies are in ‘older adults’, the majority have mean ages from 65 to 75 years, with the lower age cut-off being 60 years in most studies. This may mean that effects of age that might have been seen, for example, in 70–80 year olds, are washed out by there being relatively less effect in the lower age range. This could, of course, be overcome by studies being adequately powered to examine the effects of age in a linear fashion – possibly even over a larger age range so that the effects of age and its interaction with depression or anxiety could be examined. This would better reflect the fact that risk factors and neurobiology likely change in a linear fashion across the lifespan.^77^ Second, most studies did not analyse the effects of having early-onset compared with late-onset depression. Once again the issue is mainly one of power and as such this should be examined with sufficiently large studies. Third, a variety of paradigms have been used to study emotion processing even within similar processes. This makes it generally difficult to pool or synthesise results from different studies. Consensus on the most useful and clinically relevant paradigms would aid progress in the field. Fourth, studies have rarely attempted to determine the extent to which decline in non-emotional cognitive processes, such as executive function, may be affecting emotional processing directly. Future studies should consider undertaking testing of non-emotional memory and executive function and examining the relationship between this and emotion processing. Fifth, critically, studies have tended to be very small. Future studies should be adequately powered to show differences at least as small as 0.5 s.d. between groups. They should also, ideally, be large enough to take into account the possible effects of varying medication, late compared with early onset and age as a longitudinal factor on emotion processing. Finally, in reporting results, most studies use analysis of variance but do not report estimated marginal means and s.d., making it impossible to calculate the magnitude of differences between groups. These should be reported or effect sizes calculated.

In conclusion, the ability to correctly process and interpret emotions is an important part of social interactions, something that becomes especially important as we age. The cognitive neuropsychological hypothesis of depression also suggests that these interactions are an important part of the aetiology of depression and may provide a target for treatment. Indeed, packages of emotion recognition training are being developed to address this issue (e.g. the study by Penton-Voak et al^78^).

The review has highlighted the fact that there are relatively few large studies of emotion processing in older adults with depression and anxiety disorders. We have provided recommendations for future research.

Given the lack of studies that examine emotion processing in depression or anxiety across the life cycle, it is not possible to determine the interaction of abnormalities in these conditions with the ageing positivity bias. In general, there have been findings of a bias toward negative stimuli, and concomitant alteration of brain activity to a pattern of greater limbic and reduced prefrontal cortex activation in younger people with depression and anxiety. Similar patterns have been shown in studies in older persons, although not consistently in some aspects of emotion processing.
